# Protein stability [determination] problems

**DOI:** 10.3389/fmolb.2022.880358

**Published:** 2022-08-05

**Authors:** Faizan Ahmad

**Affiliations:** Department of Biochemistry, SCLS, Jamia Hamdard, New Delhi, India

**Keywords:** protein folding, protein stability, denaturation, denaturation mechanisms, extrapolation methods, modes of denaturation

## Abstract

Human health depends on the correct folding of proteins, for misfolding and aggregation lead to diseases. An unfolded (denatured) protein can refold to its original folded state. How does this occur is known as the protein folding problem. One of several related questions to this problem is that how much more stable is the folded state than the unfolded state. There are several measures of protein stability. In this article, protein stability is given a thermodynamic definition and is measured by Gibbs free energy change ( $\Delta G_{D}^{0}$ ) associated with the equilibrium, native (N) conformation ↔ denatured (D) conformation under the physiological condition usually taken as dilute buffer (or water) at 25 °C. We show that this thermodynamic quantity ( $\Delta G_{D}^{0}$ ), where subscript D represents transition between N and D states, and superscript 0 (zero) represents the fact that the transition occurs in the absence of denaturant, can be neither measured nor predicted under physiological conditions. However, $\Delta G_{D}$ can be measured in the presence of strong chemical denaturants such as guanidinium chloride and urea which are shown to destroy all noncovalent interactions responsible for maintaining the folded structure. A problem with this measurement is that the estimate of $\Delta G_{D}^{0}$ comes from the analysis of the plot of $\Delta G_{D}$ *versus* denaturant concentration, which requires a long extrapolation of values of $\Delta G_{D}$ , and all the three methods of extrapolation give three different values of $\Delta G_{D}^{0}$ for a protein. Thus, our confidence in the authentic value of $\Delta G_{D}^{0}$ is eroded. Another problem with this *in vitro* measurement of $\Delta G_{D}^{0}$ is that it is done on the pure protein sample in dilute buffer which is a very large extrapolation of the *in vivo* conditions, for the crowding effect on protein stability is ignored.

## Introduction

Proteins are known to affect every property that characterizes a living organism. *Sustainer of life*: Metabolic reactions which are responsible for the sustenance of life cannot occur on their own, for these are either very slow or would not occur at all. As enzymes, proteins speed up metabolic reactions. *Defense against invaders and toxic materials*: A specific defense system (antibody which is a protein) attacks specific invader (antigen). Some metabolic reactions produce toxic materials. Enzymes speed up the breakdown of such molecules into harmless molecules. *Structure and morphology*: Some of various shapes of the body parts are almost entirely due to how proteins are assembled. *Transporters of large and small molecules*: Essential large and small molecules that help to sustain life are carried from one place to other locations by proteins. *Storage of molecules and ions*: Unlike other organisms, humans do not store proteins in cells for protein synthesis or energy production. However, proteins do store small molecules and ions which are released on demand. *Transporters across membrane*: There are proteins which are involved in the movement of ions, small molecules, or macromolecules across a biological membrane. Each carrier protein is designed to recognize only one substance or one group of very similar substance. *Motility*: Movement of a whole organism or its body part(s) using metabolic energy is brought about by contraction of muscles which are made up of special proteins. To perform these and other functions, proteins adopt a specific structure (fold).

Almost all enzymes exit in a compactly folded structure under physiological conditions, usually taken as water (or dilute *neutral* buffers) at 25°C. This state of the protein is called the native state, which in practice refers to both *in vivo* and *in vitro* conditions. A few comments are however necessary. *In vivo conditions*: If the site of synthesis and site of function are the same, there is no ambiguity in the definition of the native state of the protein. However, *in vivo* definition of the native state is ambiguous in at least two cases. 1) Site of synthesis of the protein is different from the site of its action. If environmental conditions at these two sites are different and if these different conditions affect the structure of the protein differently, a question arises: Which structure should be called the native state—the state at the site of synthesis or the state at the site where protein functions? As an example, membrane proteins after their translation on the ribosome are dropped into cytoplasm which is predominantly aqueous, and then moved to the cell membrane. If such proteins are buried in the membrane, the environment there is nonpolar. The structure of membrane proteins in the cytoplasm is different from that in the membrane. A question that arises is as follows: Which should be called the native structure—the one in the cytoplasm or the one existing in the membrane? In some cases, proteins are synthesized at one location and are transported across the cell membrane, in order to reach their site of action. In order to cross the cell membrane, proteins undergo change in the structure. If this change in the structure is irreversible and subtle, a question is that which one state should be called the native state—state at the site of synthesis or state at the site where proteins function. 2) Some proteins are synthesized as preproteins, such as zymogens. *In vivo* definition of the native state of such proteins has additional problems. The structure of the processed protein (enzyme), determined by its own amino acid sequence, may be different from that present in the preprotein (zymogen). Which is the native state—that of zymogens’ or enzyme’s? As an example, pepsin is produced by removing 44 N-terminal residues of pepsinogen. *In vitro* studies have shown that pepsinogen undergoes reversible unfolding induced by urea, whereas urea-induced denaturation of pepsin is irreversible (Ahmad and McPhie, 1978a; Ahmad and McPhie, 1978b; Ahmad and McPhie, 1978c). It was concluded from these studies that the native state of the active pepsin is not determined by its own amino acid sequence. Overlaying of crystal structures of pepsin (Sielecki et al., 1990) and pepsinogen (Sielecki et al., 1991) shows that structures of the protein segment 45–326 residues of the zymogen and enzyme are identical, suggesting that functional structure of the enzyme is determined by the amino acid sequence of the zymogen. Contrary to the pepsinogen–pepsin story, the functional state of trypsin is determined by the amino acid sequence of the enzyme, for trypsin undergoes reversible denaturation (Privalov, 1979).

The native state under *in vitro* conditions: Proteins are fractionated, isolated, and purified. Studies are carried out on purified samples. Is this the native sate of the protein? There are several problems in calling this as the native state. 1) Usually harsh treatments are used during fractionation, isolation, and purification of proteins, which may cause subtle irreversible change in the structure. There is no way to compare the structure of this state with that existing in the *in vivo* conditions. 2) *In vitro* measurements are done on the pure protein sample in defining the structure and function of a protein. This is a very large extrapolation of the *in vivo* conditions, for the crowding effects on the protein structure, stability, and function are ignored. In an attempt to define the native state of a protein, the author has defined problems in defining this state. Thus, to give physiological relevance to an *in vivo* (or *in vitro*) observation, we must be aware of problems associated with the definition of the native state of proteins. Furthermore, native state and tertiary (or quaternary) structure of proteins are used interchangeably. Thus, the native structure of proteins refers to both four levels of structure (primary, secondary, tertiary, and quaternary) and four types of structure based on the secondary structure (all α-protein, all β-protein, α+β protein, and α/β protein).

If we consider proteins in aqueous environment, most of them fold. The folded structure is stabilized by various noncovalent interactions, namely, hydrophobic (Hφ) interaction, hydrogen bonding (Hb), van der Waals (vd) interactions, and salt bridge. If protein contains disulfide bond(s), its folded structure is further stabilized, for disulfide bond will destabilize the unfolded protein. The most important force that tends to destabilize the folded structure of proteins is the loss of conformational entropy of their unfolded state. A knowledge of protein stability is essential, for it is a quantity of fundamental interest in nearly all aspects of protein structure, function, and dynamics. This stability must be great enough for the protein to find and maintain its native state relative to the unfolded state, but not so great that conformational changes and adjustment are precluded, for conformational changes and adjustment are considered an integral part of proteins’ function.

There are a number of measures of stability of proteins. In the earlier literature, protein stability was often tested by subjecting a protein to high temperatures in open vessels for varying periods of time and testing for insolubility or the recovery of the activity. Although this kind of measure of stability is of great practical importance in the industry, this type of procedure depends on the irreversible process (both chemical and physical) and therefore has kinetic and equilibrium aspects. In this article, protein stability is given a thermodynamic definition. Let us consider the unfolding (denaturation) equilibrium between the native (N) state and denatured (D) state (where D state is devoid of all elements of noncovalent interactions responsible for the stability of N state) under physiological conditions (usually taken as water or dilute neutral buffer at 25°C),

N state↔D state. (1)

The protein stability ( $\Delta G_{D}^{0}$ ) is defined as the decrease in the Gibbs free energy when a denatured protein folds to give the native protein under the physiological condition, that is, $\Delta G_{D}^{0}=G_{D}^{0}-G_{N}^{0}$ , where $G_{D}^{0}$ and $G_{N}^{0}$ are Gibbs free energies of the D and N states in the absence of the denaturant, respectively. An advantage of this type of measure of protein stability is that it is the free energy of the folded native state relative to the unfolded state generated at the ribosome that drives the formation of the native protein. As a result, the thermodynamic definition, apart from the differences between the *in vivo* and *in vitro* conditions, is directly relevant to the biological process of protein folding.

More than 180 thousand crystal structures of folded proteins are known (Protein Data Bank, 1971). Some generalization from crystal structures are as follows: 1) Proteins contain secondary structure, and ∼ 2/3rd of the peptide backbone is involved in the secondary structure formation. 2) Charged groups are usually on the surface. Staggered array of positive (+) and negative (-) charges are seen. Clustering of like charges is rare. Burial of charge group is also rare. However, a few buried salt bridges are observed in many proteins. 3) Side chains with both polar (charged and uncharged) and nonpolar groups are often arranged in a manner that the charged part faces water and nonpolar part is buried. 4) Uncharged polar side chains and peptide backbone when buried are almost always involved in hydrogen bonding. 5) Nonpolar (hydrophobic) side chains are buried slightly more than 50%. However, % burial shows side chain dependence. A physical picture that emerges from examining the X-ray structures of proteins is that 1) about 85% nonpolar residues (Trp, Phe, Tyr, Ile, Leu, Val, Pro, Ala, Met, and Cys) are involved in hydrophobic interactions (Pace et al., 2014). Its average contribution to folded protein stability is about 2.33 kcal/mole-residues (Mozhaev et al., 1988). 2) About 65% of uncharged and charged polar side chains (Thr, Ser, Asn, Gln, Asp, Glu, Lys, Arg, and His) are buried (Pace et al., 2014). Its contribution to folded protein stability is about 0.54 kcal/mole-residue (Mozhaev et al., 1988). 3) There are 1.1 hydrogen bond/residue, and its contribution to protein stability is about 1 kcal/mole-bond. 4) On average, one salt bridge is buried per 100 residues (Pace et al., 2014). 5) The loss in conformational entropy during folding is very large, and its contribution to instability of the folded protein is about 3.1 ± 0.6 kcal/mole-residue (Brady and Sharp, 1997). This the most destabilizing factor. Since we know a great deal about the protein structure, and stabilizing and destabilizing forces responsible for protein stability, a question therefore arises: If the contribution of each force to the stability of folded proteins is known, can we predict protein stability in water at 25°C?

Let us consider a 100-residue long average protein (i.e., it contains 5 of each of 20 amino acids). Assume that partitioning of various forces is true in principle, and assume that model compound data are representative of all interactions in a folded protein. For the denaturation reaction (Eq. 1), one may write,

$$\Delta G_{D}^{\text{0}}=\Delta G_{H\varphi }+\Delta G_{\mathrm{vd}}+\Delta G_{\mathrm{psc}}+\Delta G_{\mathrm{Hb}}+\Delta G_{\mathrm{ele}}+\Delta G_{\mathrm{conf}},$$ (2)

where $\Delta G_{H\varphi }$ is the contribution due to hydrophobic interaction, $\Delta G_{\mathrm{vd}}$ is the contribution due to van der Waals interaction, $\Delta G_{\mathrm{psc}}$ is the contribution due to buried polar side chain (psc), $\Delta G_{\mathrm{Hb}}$ is the contribution due to buried hydrogen bonding, $\Delta G_{\mathrm{ele}}$ is the contribution due to buried salt bridge, and $\Delta G_{\mathrm{conf}}$ is the contribution due to loss in conformational entropy. The buried nonpolar side chains contribute to protein stability in two ways: first contribution comes from the removal of nonpolar side chains from water to protein interior and second contribution comes from the tight packing of nonpolar side chains giving rise to van der Waals interaction. Hence, $\left(\Delta G_{H\varphi }+\Delta G_{\mathrm{vd}}\right)$ term in Eq. 2 is estimated from the transfer-free energy from water to nonpolar solvent. An average value of - 2.33 kcal/mole-nonpolar residue is reported (Mozhaev et al., 1988); 85% of these buried side chains would stabilize folded protein. Thus, for the protein in question, $\left(\Delta G_{H\varphi }+\Delta G_{\mathrm{vd}}\right)$ contribution is 99 kcal/mole protein (= 0.85 × 50 × 2.33). $\Delta G_{\mathrm{psc}}$ contribution to stability is also estimated from the transfer-free energy of the polar amino acid side chains from water to organic solvent, and the average value of + 0.54 kcal/mole-polar side chain is reported earlier (Mozhaev et al., 1988). Thus, for the query protein, $\Delta G_{\mathrm{psc}}$ contribution is 16 kcal/mole protein (= 0.65 × 45 × 0.54). The protein in question would contain 110 hydrogen bonds, and each buried hydrogen bond contributes 1 kcal to protein stability. Thus, $\Delta G_{\mathrm{Hb}}$ contribution would be 110 kcal/mole protein. For the query protein, values of $\Delta G_{\mathrm{ele}}$ and $\Delta G_{\mathrm{conf}}$ are + 5 and 310 (= 100 × 3.1) kcal/mole protein, respectively. Substituting for each contribution in Eq. 2, we get a value of - 80 (= + 99 + 16 + 110 + 5–310) kcal/mole protein. This means that a randomly selected 100-residue long polypeptide will likely not fold into a stable structure in water at 25°C (see Eq. 1). On the contrary, most natural proteins are evolved to fold in water at 25°C. This apparent paradox is most probability due to the fact that model compounds’ data are inaccurate, and they will remain so, for these interactions are extremely model- and solvent-dependent. Thus, this method cannot be used to estimate protein stability. The author calls this as protein stability determination problem number 1.

$\Delta G_{D}^{0}$ values of almost all well-characterized folded proteins that have had their stability measured lie in the range 5–15 kcal/mole under physiological conditions (Ali, 2016). Hence, $K_{D}^{0}$ (equilibrium constant for the denaturation process given by Eq. 1), defined as $K_{D}^{0}=\left[D\right]/\left[N\right]$ (where [D] and [N] are molar concentrations of the denatured and native molecules, respectively), can be determined if concentrations of N and D molecules are known. If $\Delta G_{D}^{0}$ is 5 kcal/mole, then *K*_D_^0^ { = Exp (- ∆*G*_D_^0^/*RT*) = Exp (-5,000/1.9872 × 298.2) = 2.2 × 10^−4^} is ∼10^–4^. If $\Delta G_{D}^{0}$ is 15 kcal/mole, then *K*_D_^0^ { = Exp (- ∆*G*_D_^0^/*RT*) = Exp (-15000/1.9872 × 298.2) = 1.0 × 10^−11^} is 10^–11^, that is, the ratio ([D]/[N]) is in the range 10^–4^—10^–11^. This means that at the most, there is only one D molecule out of 10 thousand—10 trillion protein molecules. These considerations show that concentration of D molecule is far too low to be detected by conformational techniques. Hence, equilibrium between N and D states and hence $\Delta G_{D}^{0}$ cannot be measured by these techniques under physiological condition (in water (or dilute buffer) at 25°C). This is the $\Delta G_{D}^{0}$ determination problem number 2.

The measurement of D state is possible in the presence of denaturant. This means that equilibrium between N and D states (Eq. 1) can be measured in the presence of appropriate concentrations of the denaturant. It is therefore said that the measurement of stability of a protein ( $\Delta G_{D}^{0}$ ) is connected to the study of protein denaturation. There are many modes of denaturation. Physical modes are heat and pressure. Chemical modes are pH, urea, guanidinium chloride (GdmCl), lithium salts, CaCl_2_, detergents, and mixtures of denaturants. It has been observed that for most proteins, urea- and GdmCl-induced denatured state is maximally unfolded, whereas heat, pH, lithium, and calcium salts give partial denatured states (Tanford, 1968; Ahmad and Salahuddin, 1974; Lananje, 1978; Ahmad and Bigelow, 1979; Ahmad, 1983; Ahmad, 1984; Ahmad, 1991; Singh et al., 2015).

### Denaturation curve

**FIGURE 1:**
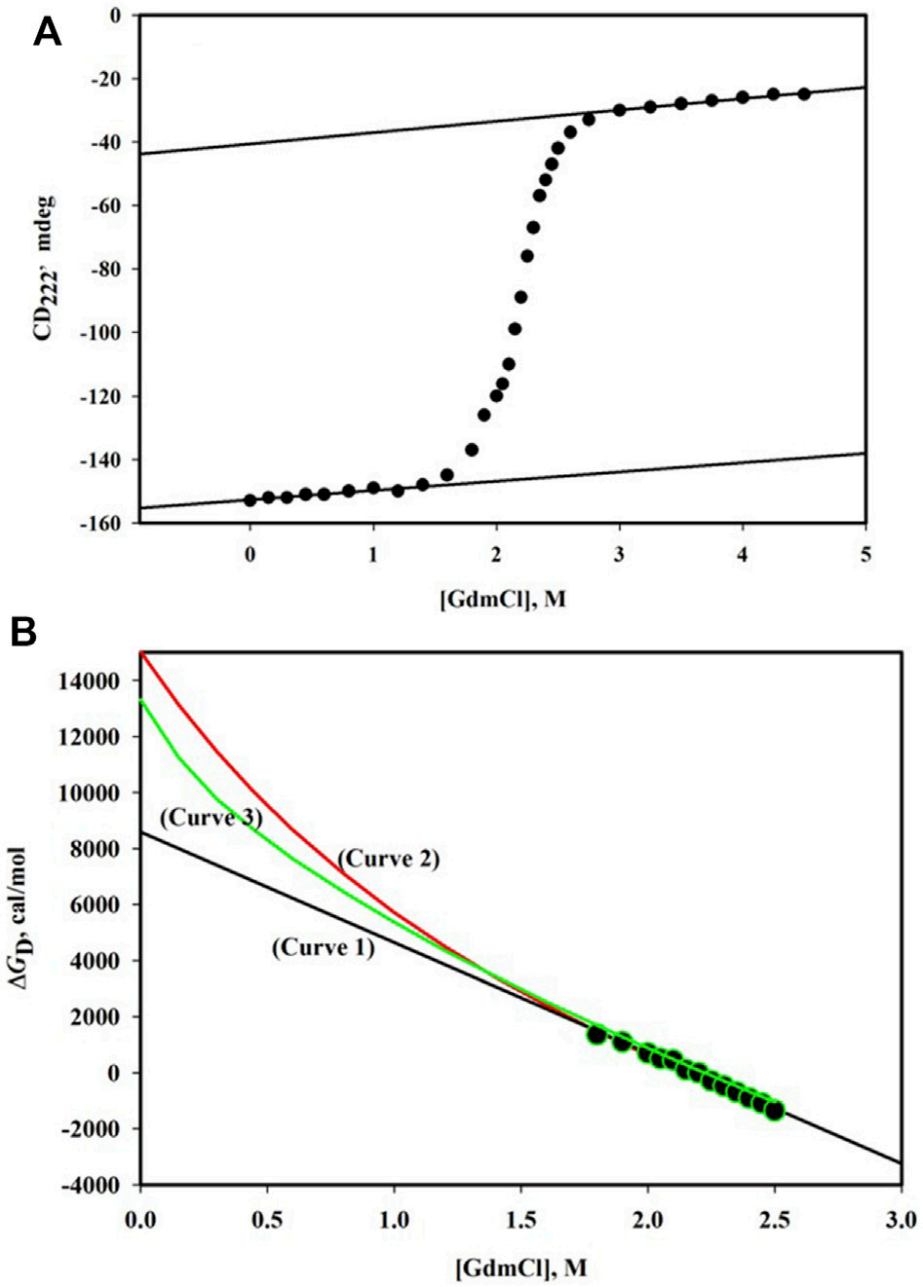
**(A)** GdmCl-induced denaturation curve monitored by CD_222_ of α-phycoerythrin at pH 7.0 and 25°C. The protein concentration was 0.75 mg/ml and path length of the cell was 0.1 cm. The linear dependence of the pre-transition baseline is described by the relation, CD_222_ = -152.6 + 2.90 [GdmCl]. The linear dependence of the post-transition baseline is described by the relation, CD_222_ = -40.5 + 3.57 [GdmCl]. **(B)** Plot of Δ*G*_D_ *versus* [GdmCl]. Observed data (filled circles) were analyzed using three models of denaturation (see text). The best fit parameters are as follows: Δ*G*_D_^0^ = 8.58 (±0.20) kcal/mole and *m* = 3.94 (±0.09) kcal/mole. M for the linear model (curve 1); Δ*G*_D_^0^ = 15.03 (±0.46) kcal/mole, Δn = 48 (±1), and *k* = 0.7 for the binding model (curve 2); and Δ*G*_D_^0^ = 12.90 (±0.14) kcal/mole and Δα = 0.31 (±0.01) for the transfer-free energy model.

GdmCl-induced denaturation of proteins is followed by observing changes in their physical properties which are significantly different for N and D states. Figure 1A shows the denaturation curve of CD_222_, the far-UV circular dichroism (CD) signal at 222 nm, for the α-phycoerythrin subunit. This denaturation curve is traditionally divided into three regions—pre-transition region ([GdmCl], the molar concentration of GdmCl) is in the range 0 M - < 1.6 M, post-transition region ([GdmCl] > 2.8 M), and transition region (1.6 M < [GdmCl] < 2.8 M). It has been observed that this protein undergoes reversible denaturation induced by GdmCl at pH 7.0 and 25°C.

### Analysis of denaturation curve

The denaturation curve shown in Figure 1A can be analyzed for the estimation of protein stability ( $\Delta G_{D}^{0}$ ), if the protein operates under following constraints. 1) Structure of the protein in N and D states is independent of protein concentration throughout the denaturant concentration range. 2) Structure of protein in the N and D states does not depend on [GdmCl]. This is why any change in the optical property in the pre- and post-transition regions is called solvent effect. This solvent effect is assumed to be linear in the denaturant concentration ([GdmCl]). Recently, this assumption is dealt in detail elsewhere (Lindorff-Larsen and Teilum, 2021). 3) Denaturation (Eq. 1) is a two-state process; equilibrium constant, *K*_D_, in the transition region can be estimated as a function of [GdmCl].

If $f_{N}$ and $f_{D}$ represent fractions of the protein in N and D states, respectively, then for a two-state denaturation, $f_{N}+f_{D}=1$ . The observed property ( $y_{\mathrm{obs}}$ ) used to monitor denaturation (e.g., here, CD_222_) at any point on the transition curve (Figure 1A) is given by the relation,

$$y_{\mathrm{obs}}=f_{N}y_{N}+f_{D}y_{D},$$ (3)

where $y_{N}$ and $y_{D}$ are properties of the protein at the same denaturant concentration at which $y_{\mathrm{obs}}$ has been measured. By definition, *K*_D_ is given by the relation,

$$K_{D}=\left(f_{D}/f_{N}\right)=\left(f_{D}/1-f_{D}\right),$$ (4)

Using Eq. 3, one writes

$$K_{D}=\left(y_{\mathrm{obs}}-y_{N}\right)/\left(y_{D}-y_{\mathrm{obs}}\right),$$ (5)

$$\Delta G_{D}=-RT\ln K_{D},$$ (6)

where *T* is the temperature in kelvin (K) and *R* is the universal gas constant (= 1.9872 cal/mol. K). Eq. 5 is used to determine $K_{D}$ in the presence of different concentrations of GdmCl in the transition region. It should be noted that values of $y_{N}$ and $y_{D}$ at a given [GdmCl] in the transition region are obtained from the linear extrapolation of pre- and post-transition baselines. Only those values of $K_{D}$ are used to estimate values of $\Delta G_{D}$ (Eq. 6) for which $f_{D}$ is in the range 0.1 < *f*_D_ < 0.9. Thus, we can measure $\Delta G_{D}$ accurately only in the presence of a denaturant. However, Δ*G*_D_^0^ (protein stability) is defined as the value of $\Delta G_{D}$ in the absence of the denaturant.

## Results and discussion

Figure 1B shows the plot of Δ*G*_D_ *versus* [GdmCl]. An extrapolation of Δ*G*_D_ to 0 M of the denaturant will yield protein stability. However, for any extrapolation, we need to have a model that answers the question: Why does a protein get denatured in the presence of the denaturant? There are three models of denaturation (Schellman, 1978; Ahmad, 1991). *Linear-free energy* model, which has been proven on thermodynamic grounds (Schellman, 1978), states that for dilute protein solution in the presence of the denaturant, $\Delta G_{D}$ varies linearly with [denaturant], that is, for a two-state transition,

$$\Delta G_{D}=\Delta G_{D}^{0}-m\left[\mathrm{denaturant}\right],$$ (7)

where *m* gives the dependence of Δ*G*_D_ on [denaturant], that is, *m* = (δΔ*G*_D_/δ[denaturant])_*T,P,pH*_, a measure of cooperativity of the denaturation. A linear least-squares analysis according to Eq. 7 gave a value of 8.58 kcal/mol for Δ*G*_D_^0^ (see curve 1in Figure 1B).

The *binding* model (Tanford, 1970) is also used to analyze data (Δ*G*_D_, [GdmCl]) shown in Figure 1B. According to this model, there exist *specific* binding sites on the protein for the denaturant. If it is assumed 1) that all binding sites are independent of the extent of binding at other sites, 2) that all binding constants for each state are equal, and 3) that the denaturant binds both native and denatured protein with an identical value, then dependence of $\Delta G_{D}$ on *a*, the activity of the denaturant, is given by the relation,

$$\Delta G_{D}=\Delta G_{D}^{0}-\Delta nRT\ln\left(1+ka\right),$$ (8)

where Δn is the number of the newly exposed binding sites on denaturation and *k* is the specific binding constant. In the case of GdmCl, *a* is replaced by a_±_, the mean ion activity (= √*a*_GdmCl_). The relation between a_±_ and [GdmCl] is given by Pace (Pace, 1986). The analysis of $\Delta G_{D}$ according to Eq. 8 yields a value of 15.03 kcal/mole for $\Delta G_{D}^{0}$ (see curve 2 in Figure 1B), which is significantly larger than the value when the same data set was analyzed using Eq. 7.

The third model used to determine $\Delta G_{D}^{0}$ of proteins is the *transfer-free energy model* which uses a thermodynamic cycle to describe the processes of denaturation in the absence and presence of a denaturant (Tanford, 1970). $\Delta G_{D}$ of the protein is described in terms of transfer-free energy of protein groups (amino acid side chains and peptide backbone), which is given by the relation (Pace, 1975; Ahmad and Bigelow, 1986),

$$\Delta G_{D}=\Delta G_{D}^{0}-\Delta \alpha \sum \left(n_{i}\delta {\text{g}}_{\mathrm{tr}\text{,}i}\right),$$ (9)

where Δα is the average fractional change in the accessibility of all protein groups, ${\text{n}}_{i}$ is the total number of the *i*th kind of the group, and $\delta {\text{g}}_{\mathrm{tr}\text{,}i}$ is the transfer-free energy of the *i*th kind of the group from water to a given concentration of the denaturant. $\delta {\text{g}}_{\mathrm{tr}\text{,}i}$ values of each amino acid side chain and a peptide group were obtained from the solubility measurements of free amino acids, diglycine and triglycine. These values are compiled by Pace (Pace, 1975), and their dependence on [GdmCl] are given elsewhere (Ahmad and Bigelow, 1982). The analysis of the observed Δ*G*_D_ values according to Eq. 9 gave a value of 13.22 kcal/mol for $\Delta G_{D}^{0}$ (see curve 3 in Figure 1B), which is larger than the value obtained from the linear extrapolation method but smaller than that obtained from the extrapolation according to the binding model.

It has been observed that all the methods of extrapolation (Eqs 7-9) fit the same set of (Δ*G*_D_ [GdmCl]) data equally well (see Figure 1B). Thus, the discrepancy between estimates of $\Delta G_{D}^{0}$ from three different methods of extrapolation of the same set of (Δ*G*_D_, [GdmCl]) data for a protein erodes our confidence in this stability parameter and leaves us to ask a question: Which extrapolation method gives an authentic value of $\Delta G_{D}^{0}$ ? This is protein stability determination problem number 3.

The linear extrapolation method has been justified on theoretical (Schellman, 1978) and experimental (Ahmad and Bigelow, 1982; Pace, 1986; Santro and Bolen, 1988; Ahmad et al., 1994; Gupta et al., 1996; Gupta and Ahmad, 1999) grounds. Furthermore, hydrogen exchange measurements provide a direct estimate of Δ*G*_D_^0^ under native conditions (in the absence of any denaturant). These measurements also support the linear extrapolation method (Bai et al., 1994; Huyghes-Despointe et al., 1999; Huyghes-Despointe et al., 2001). It has been argued that since [denaturant] is very high in the transition region, binding of the denaturant to proteins cannot be specific; it could rather be a forced binding (Schellman, 1978). On this ground, the binding model may be rejected. The transfer-free energy model treats proteins as solution of free amino acids and peptide (—NH—CH—CO—). The steric bulk of peptide backbone reduces the number of solvent molecules in contact with the exposed side chain and *vice versa* (Nemethy, 1967). Thus, the unfolded state of the protein cannot be regarded as dilute solution of the constituent groups (Privalov, 1979). As argued earlier (Ahmad and Bigelow, 1986), the extrapolation method (Eq. 9), which uses $\delta {\text{g}}_{\mathrm{tr}\text{,}i}$ values for free amino acids and peptide group, cannot be justified to analyze denaturation curves for the estimation of $\Delta G_{D}^{0}$ .

Assuming that one can estimate *in vitro* protein stability using linear-free energy model, but this estimate is on the isolated protein. In contrast to this *in vitro* condition, a protein exists in crowded environment (100–500 mg/ml of macromolecules) in the cell (Feig et al., 2017). Artificial crowding in the test tube has been shown to influence protein function and protein stability (Shahid et al., 2017). Methods for direct measurements of $\Delta G_{D}^{0}$ *in vivo* are developed. Technical challenges to measure this thermodynamic quantity in the living cells are extremely difficult. Attempts have been made by only a few groups who reported *in vivo* stability of proteins. A recent study has reviewed the work relating to the *in vivo* protein stability measurements (Danielsson and Oliveberg, 2017). It has been observed that protein stability in the cell increases (Dhar et al., 2011; Monteith and Pielak, 2014) for some proteins and decreases (Ignatova and Gierasch, 2004; Ignatova et al., 2007; Ignatova and Gierasch, 2009) for some other proteins as compared to the respective *in vitro* value.

## Conclusion

In the absence of an experimental evidence for the presence of specific binding site(s) on the protein for the chemical denaturant, the binding model should be abandoned. A problem with the transfer-free energy model, which uses a thermodynamic cycle to describe the processes of denaturation in the absence and presence of a denaturant, is that there is no accurate method to determine transfer-free energy of protein groups when they are attached to the polypeptide chain. The linear-extrapolation method, which has been justified on theoretical and experimental grounds, should be used to obtain $\Delta G_{D}^{0}$ of proteins operating under certain constraints.

## Data Availability

The original contributions presented in the study are included in the article/Supplementary Material; further inquiries can be directed to the corresponding author.
